# Differential CpG methylation at *Nnat* in the early establishment of beta cell heterogeneity

**DOI:** 10.1007/s00125-024-06123-6

**Published:** 2024-03-21

**Authors:** Vanessa Yu, Fiona Yong, Angellica Marta, Sanjay Khadayate, Adrien Osakwe, Supriyo Bhattacharya, Sneha S. Varghese, Pauline Chabosseau, Sayed M. Tabibi, Keran Chen, Eleni Georgiadou, Nazia Parveen, Mara Suleiman, Zoe Stamoulis, Lorella Marselli, Carmela De Luca, Marta Tesi, Giada Ostinelli, Luis Delgadillo-Silva, Xiwei Wu, Yuki Hatanaka, Alex Montoya, James Elliott, Bhavik Patel, Nikita Demchenko, Chad Whilding, Petra Hajkova, Pavel Shliaha, Holger Kramer, Yusuf Ali, Piero Marchetti, Robert Sladek, Sangeeta Dhawan, Dominic J. Withers, Guy A. Rutter, Steven J. Millership

**Affiliations:** 1 Department of Metabolism, Digestion and Reproduction, Faculty of Medicine, Imperial College London, London, UK; 2 Lee Kong Chian School of Medicine, Nanyang Technological University, Singapore, Republic of Singapore; 3 MRC Laboratory of Medical Sciences, London, UK; 4 Quantitative Life Sciences Program, McGill University, Montréal, QC Canada; 5 Department of Computational and Quantitative Medicine, Beckman Research Institute, City of Hope, Duarte, CA USA; 6 Department of Translational Research and Cellular Therapeutics, Arthur Riggs Diabetes and Metabolism Research Institute, City of Hope, Duarte, CA USA; 7 Present Address: Biomedical Research Centre, School of Biological Sciences, University of East Anglia, Norwich, UK; 8 Department of Clinical and Experimental Medicine, and AOUP Cisanello University Hospital, University of Pisa, Pisa, Italy; 9 Present Address: Medical Sciences Division, University of Oxford, Oxford, UK; 10 CHUM Research Center and Faculty of Medicine, University of Montréal, Montréal, QC Canada; 11 Institute of Clinical Sciences, Faculty of Medicine, Imperial College London, London, UK; 12 Present Address: Imaging Resource Facility, Research Operations, St George’s, University of London, London, UK; 13 Nutrition, Metabolism and Health Programme & Centre for Microbiome Medicine, Lee Kong Chian School of Medicine, Nanyang Technological University Singapore, Singapore, Republic of Singapore; 14 Singapore Eye Research Institute (SERI), Singapore General Hospital, Singapore, Republic of Singapore; 15 Clinical Research Unit, Khoo Teck Puat Hospital, National Healthcare Group, Singapore, Republic of Singapore; 16 Departments of Medicine and Human Genetics, McGill University, Montréal, QC Canada

**Keywords:** Beta cell development, Ca^2+^, Connectivity, CpG methylation, Heterogeneity, Identity, Imprinted genes, Insulin, Islet, Neuronatin, Type 2 diabetes

## Abstract

**Aims/hypothesis:**

Beta cells within the pancreatic islet represent a heterogenous population wherein individual sub-groups of cells make distinct contributions to the overall control of insulin secretion. These include a subpopulation of highly connected ‘hub’ cells, important for the propagation of intercellular Ca^2+^ waves. Functional subpopulations have also been demonstrated in human beta cells, with an altered subtype distribution apparent in type 2 diabetes. At present, the molecular mechanisms through which beta cell hierarchy is established are poorly understood. Changes at the level of the epigenome provide one such possibility, which we explore here by focusing on the imprinted gene *Nnat* (encoding neuronatin [NNAT]), which is required for normal insulin synthesis and secretion.

**Methods:**

Single-cell RNA-seq datasets were examined using Seurat 4.0 and ClusterProfiler running under R. Transgenic mice expressing enhanced GFP under the control of the *Nnat* enhancer/promoter regions were generated for FACS of beta cells and downstream analysis of CpG methylation by bisulphite sequencing and RNA-seq, respectively. Animals deleted for the de novo methyltransferase DNA methyltransferase 3 alpha (DNMT3A) from the pancreatic progenitor stage were used to explore control of promoter methylation. Proteomics was performed using affinity purification mass spectrometry and Ca^2+^ dynamics explored by rapid confocal imaging of Cal-520 AM and Cal-590 AM. Insulin secretion was measured using homogeneous time-resolved fluorescence imaging.

**Results:**

*Nnat* mRNA was differentially expressed in a discrete beta cell population in a developmental stage- and DNA methylation (DNMT3A)-dependent manner. Thus, pseudo-time analysis of embryonic datasets demonstrated the early establishment of *Nnat*-positive and -negative subpopulations during embryogenesis. NNAT expression is also restricted to a subset of beta cells across the human islet that is maintained throughout adult life. NNAT^+^ beta cells also displayed a discrete transcriptome at adult stages, representing a subpopulation specialised for insulin production, and were diminished in *db/db* mice. ‘Hub’ cells were less abundant in the NNAT^+^ population, consistent with epigenetic control of this functional specialisation.

**Conclusions/interpretation:**

These findings demonstrate that differential DNA methylation at *Nnat* represents a novel means through which beta cell heterogeneity is established during development. We therefore hypothesise that changes in methylation at this locus may contribute to a loss of beta cell hierarchy and connectivity, potentially contributing to defective insulin secretion in some forms of diabetes.

**Data availability:**

The mass spectrometry proteomics data have been deposited to the ProteomeXchange Consortium via the PRIDE partner repository with the dataset identifier PXD048465.

**Graphical Abstract:**

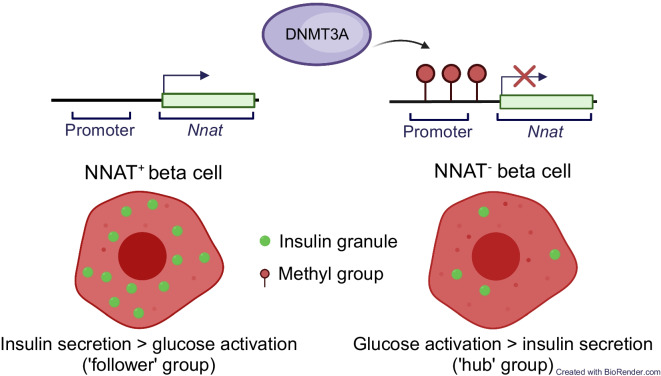

**Supplementary Information:**

The online version contains peer-reviewed but unedited supplementary material available at 10.1007/s00125-024-06123-6.


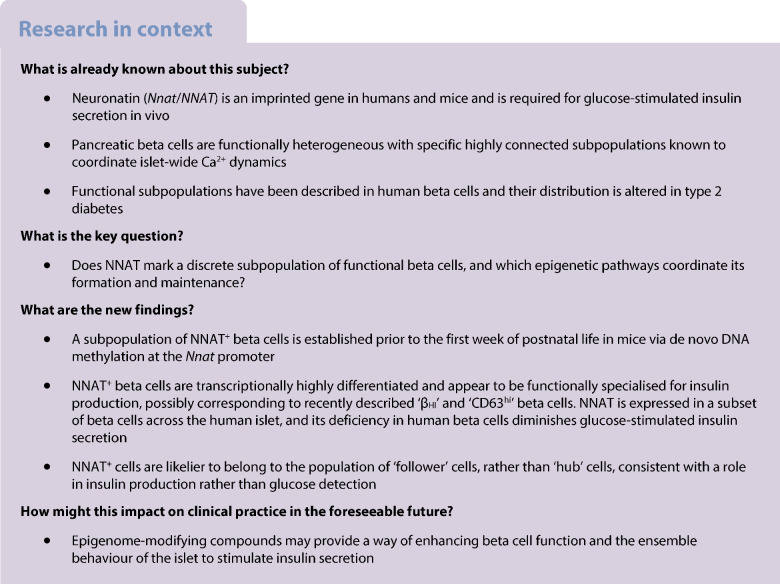


## Introduction

Insulin-producing beta cells are central to the modulation of glucose homeostasis, and their impaired function, loss of identity or lowered numbers result in type 2 diabetes [1]. Previous studies have provided an understanding of the transcriptional machinery that orchestrates beta cell development from early pancreatic and endocrine precursors [2]. To bolster these transcriptional programmes in vivo, chronic regulation via the epigenome appears to step in to maintain beta cell identity in the long term [3–7].

Early reports [8, 9], and more recent studies based on single-cell transcriptomic profiling [10–12], electrophysiology [13] and functional imaging [14–17], have demonstrated functional heterogeneity amongst individual beta cells (reviewed in [18]). Identified subpopulations have been associated with known markers or maturation states (*Flattop*/*Cfap126* [16], polysialyated-neural cell adhesion molecule [PSA-NCAM] [19], CD81 [20], CD24 [7, 21], tyrosine hydroxylase [TH] [6], neuropeptide Y (NPY) [22], CD63 [23]), are ‘virgin’ beta cells [24] or are defined by their roles in coordinating islet-wide Ca^2+^ dynamics (e.g. ‘hubs’ [14], ‘leaders’ [15, 25] and ‘first responders’ [26]). Furthermore, loss of beta cell heterogeneity or intercellular connectivity may contribute to the development of type 2 diabetes [14, 15, 27]. Importantly, functional subpopulations have also been demonstrated in human beta cells [11, 12, 28], and the distribution of antigenically defined sub-groups (based on CD9 and ST8 α-*N*-acetylneuraminide α-2,8-sialyltransferase [ST8SIA1] positivity) is altered in type 2 diabetes [10]. The features underlying beta cell heterogeneity include pathways governing glucose sensing and metabolism [14–16], insulin content and secretory competence [5, 13, 24, 29, 30], and cilia activity and localisation within the islet [25]. Recently, two discrete populations of ‘CD63^hi^’ and ‘CD63^lo^’ cells have been described [23], with ‘CD63^hi^’ cells enriched for CD63 and for insulin content and glucose-stimulated insulin secretion (GSIS). Epigenomically defined (by histone methylation, H3K27me3) CD24-positive ‘β_HI_’ beta cells with enhanced insulin content and GSIS [7] may partly overlap the ‘CD63^hi^’ population [23].

Imprinted genes are expressed from a single allele in a parent-of-origin-specific manner and their expression is also controlled via epigenetic modifications, notably DNA methylation. Imprinted genes often play key physiological roles, particularly in early (fetal and postnatal) growth and development, controlling a wide range of cellular processes. Thus, human imprinting disorders involving altered expression from specific imprinted loci are associated with severe childhood developmental and metabolic complications (reviewed in [31]).

Imprinted genes play key functional roles in pancreatic beta cells by modulating insulin secretory machinery or beta cell mass [32]. Correspondingly, imprinted gene expression is dysregulated both in beta cells with diminished GSIS and in pancreatic islets from individuals with type 2 diabetes, and single nucleotide polymorphisms (SNPs) at imprinted loci are associated with type 2 diabetes risk [18]. Monoallelic expression of imprinted genes is maintained trans-generationally by differential methylation between parental alleles at imprinted loci in the germline [33], with additional ‘somatic’ or ‘secondary’ differentially methylated regions (DMRs) also established post fertilisation [34].

We have previously shown [35] that the paternally expressed, imprinted gene *Nnat* (encoding neuronatin [NNAT]) is nutrient-regulated in pancreatic beta cells, and controls insulin content and GSIS by modulating early insulin precursor processing at the signal peptidase complex (SPC) [35]. At extra-pancreatic sites, changes in *Nnat* expression also modulate appetite and metabolism [36–38]. Here, we explore the possibility that differential methylation of the *Nnat* gene contributes to the functional heterogeneity of embryonic and adult pancreatic beta cells.

## Methods

For details, please refer to electronic supplementary material (ESM) Methods.

### Bioinformatic clustering analysis

Single-cell RNA-seq (scRNA-seq) embryonic and adult islet datasets [5, 39–42] were assessed for hypervariability of gene expression and unbiased clustering using the scran v1.28.2 (https://bioconductor.org/packages/release/bioc/html/scran.html) and RaceID v0.3.0 (https://cran.r-project.org/web/packages/RaceID/index.html) packages, respectively, as described in the ESM Methods.

### Pseudo-time analysis

scRNA-seq datasets [39] were processed with Seurat v4.3 and clustered and processed for pseudo-time analysis, followed by integration with a dataset from adult mouse islets [41, 42], according to the ESM Methods.

### Study approval

All animal procedures were in accordance with the UK Animals (Scientific Procedures) Act 1986 and approved by the UK Home Office (Project licence (PPL) number: PP4712156). All animal studies have been approved by the Imperial College Animal Welfare and Ethical Review Body.

### Animal models

Transgenic mouse lines expressing *Cre* recombinase under the control of the rat insulin promoter (RIP) [43] and with a tdTomato reporter downstream of a stop codon flanked by *loxP* sites [44] have been described previously. Mice with *Nnat*-driven enhanced GFP (eGFP) expression were purchased from the Mutant Mouse Resource and Research Centre (MMRRC, USA) repository [Tg(*Nnat-*EGFP)EA106Gsat/Mmucd, stock no. 010611-UCD]. Mice with global deletion of *Nnat* [35] and conditional *Dnmt3a* null mice generated by crossing to *Pdx1*-*Cre* are described in the ESM Methods. *db/db* and control C57BLKS/J mice on the BKS background were obtained from The Jackson Laboratory, USA (JAX 000642 and JAX 000662, respectively).

### Primary islet isolation and FACS

To isolate primary islets, the pancreas was inflated with Liberase TM (Roche, UK), digested at 37°C and purified using a Histopaque 1119/1083/1077 gradient (Sigma-Aldrich, UK), and islets were hand-picked. For FACS-based experiments, cells from purified islets were isolated using Accutase (Sigma-Aldrich) and sorted using a FACSAria III flow cytometer (BD Biosciences, UK), as detailed in the ESM Methods. Total insulin content was assessed using ultra-sensitive insulin homogeneous time-resolved fluorescence (HTRF) assay kits (Cisbio, France).

### Intracellular calcium imaging

Pancreatic islets from reporter mice expressing *Nnat*-eGFP were isolated as above, with Ca^2+^ imaging of whole islets performed after loading the cytosol with 2 μmol/l Cal-590 AM (Stratech, UK). Images were captured on an Axiovert microscope (Zeiss, Germany) equipped with a ×10, 0.3–0.5 numerical aperture (NA) objective and a ImagEM camera (Hamamatsu, Japan) coupled to a Nipkow spinning-disk head (CSU-10, Yokogawa, Japan) and illuminated at 490 nm or 530 nm.

For experiments involving global *Nnat* null mice [35], islets were incubated with 4.5 μmol/l Cal-520 AM (Stratech), and imaging was performed on a Nikon (Japan) Eclipse Ti microscope equipped with a ×40/1.2 NA oil objective and an ibidi heating system. Cal-520 AM was excited with a 491 nm laser line and emitted light filtered at 525/50 nm. Images were acquired with an ORCA-Flash 4.0 camera (Hamamatsu) and Metamorph software (Molecular Devices). Pearson-based connectivity and correlation analyses in an imaged islet were performed with Ca^2+^ signals smoothed, binarised and analysed as described in the ESM Methods.

### Histological techniques and immunofluorescence

Dissected tissues were fixed, cryoprotected and embedded in optimal cutting temperature (OCT) and stored at −80°C. Sections (10 μm) were cut using a CM1950 Cryostat (Leica, Germany) and immunostained using primary and secondary antibodies (listed in the ESM Methods), and imaged using a TCS SP5 confocal microscope (Leica). Immunostaining of paraffin-embedded pancreas from mice with beta cell-selective deletion of *Dnmt3a* was performed according to references [6, 22] as described in the ESM Methods. Human pancreases were processed, immunostained and imaged as described in the ESM Methods with the approval of the Ethics Committee of the University of Pisa, upon written consent of donors’ next-of-kin.

### Cell culture, RNA silencing and GSIS

Human EndoC-βH1 and rat INS1E beta cells were cultured as previously described [35] and incubated with lentiviruses expressing an *NNAT*-targeting short hairpin RNA (shRNA; Sigma-Aldrich) or Silencer Select siRNAs (Ambion, USA), respectively, as described in the ESM Methods. GSIS assays were performed using ultra-sensitive insulin HTRF assay kits (Cisbio) for insulin quantification.

### Immunoprecipitation and mass spectrometry

Cells were processed for immunoprecipitation and incubated with antibodies against NNAT (ab27266, Abcam, UK, 1:500) prior to mass spectrometry analysis [35] as described in the ESM Methods.

### Western immunoblotting, RT-PCR, RNA-seq

For western blotting, EndoC-βH1 cells were processed as previously described [35] using primary antibodies against NNAT (ab27266, Abcam, 1:2000) and β-tubulin (clone 9F3, stock no. 2128, Cell Signaling, USA, 1:5000). For RT-PCR, mRNA was purified using Allprep or RNeasy kits (both Qiagen, Germany) and reverse transcribed, and cDNA was assessed by quantitative RT-PCR using Taqman reagents (all Life Technologies, USA) on a QuantStudio 7 Real Time PCR cycler.

For RNA-seq, RNA from FACS-purified cells was quantified and assessed for integrity using a Bioanalyzer 2100 and an RNA 6000 Pico assay (Agilent Technologies). RNA was processed using a NEBNext Ultra II Directional RNA Library Prep Kit for Illumina paired with a Poly(A) mRNA magnetic isolation module and AMPure XP SPRIselect beads (Beckman Coulter, UK). Libraries were sequenced using a NextSeq 500 High Output sequencer (Illumina, USA), with 2×75 bp length at 50 million reads per sample. Further details can be found in the ESM Methods.

### Bisulphite sequencing

Genomic DNA was extracted from FACS-purified cells using Allprep DNA/RNA/protein mini kits from Qiagen. Bisulphite sequencing experiments were performed using an EZ DNA Methylation-Gold kit from Zymo Research, USA, an EpiTaq hot start kit (TaKaRa Bio, Japan) and a CloneJET PCR cloning kit (Life Technologies), with DNA purified using a Wizard SV 96 plasmid system (Promega, USA) before Sanger sequencing (Genewiz, now Azenta Life Sciences, UK), and then analysed using bisulphite sequencing DNA methylation analysis (BISMA) software.

### Statistical analysis

Data are shown as mean±SEM in all figure panels. Data were assessed using GraphPad Prism 9.0, with <5% error probability considered significant (i.e. *p*<0.05). Further statistical information, such as *n* numbers and *p* values, are provided in the figure legends.

## Results

### NNAT expression is heterogeneous across the islet and represents a highly differentiated beta cell subtype

We have previously shown that *Nnat* is crucial for insulin storage and GSIS in the mouse [35]. NNAT expression is diminished in rodent models of obesity and diabetes including the Zucker diabetic fatty (ZDF) rat model (85% decrease, *p*=0.0023, *n*=5 [45]) and obese *ob/ob* mice (ESM Fig. 1a). Pancreatic sections of *db/db* mice displayed a loss of NNAT immunoreactivity across islets, and a significant reduction of NNAT^+^ beta cells (ESM Fig. 1b,c).

**Fig. 1:**
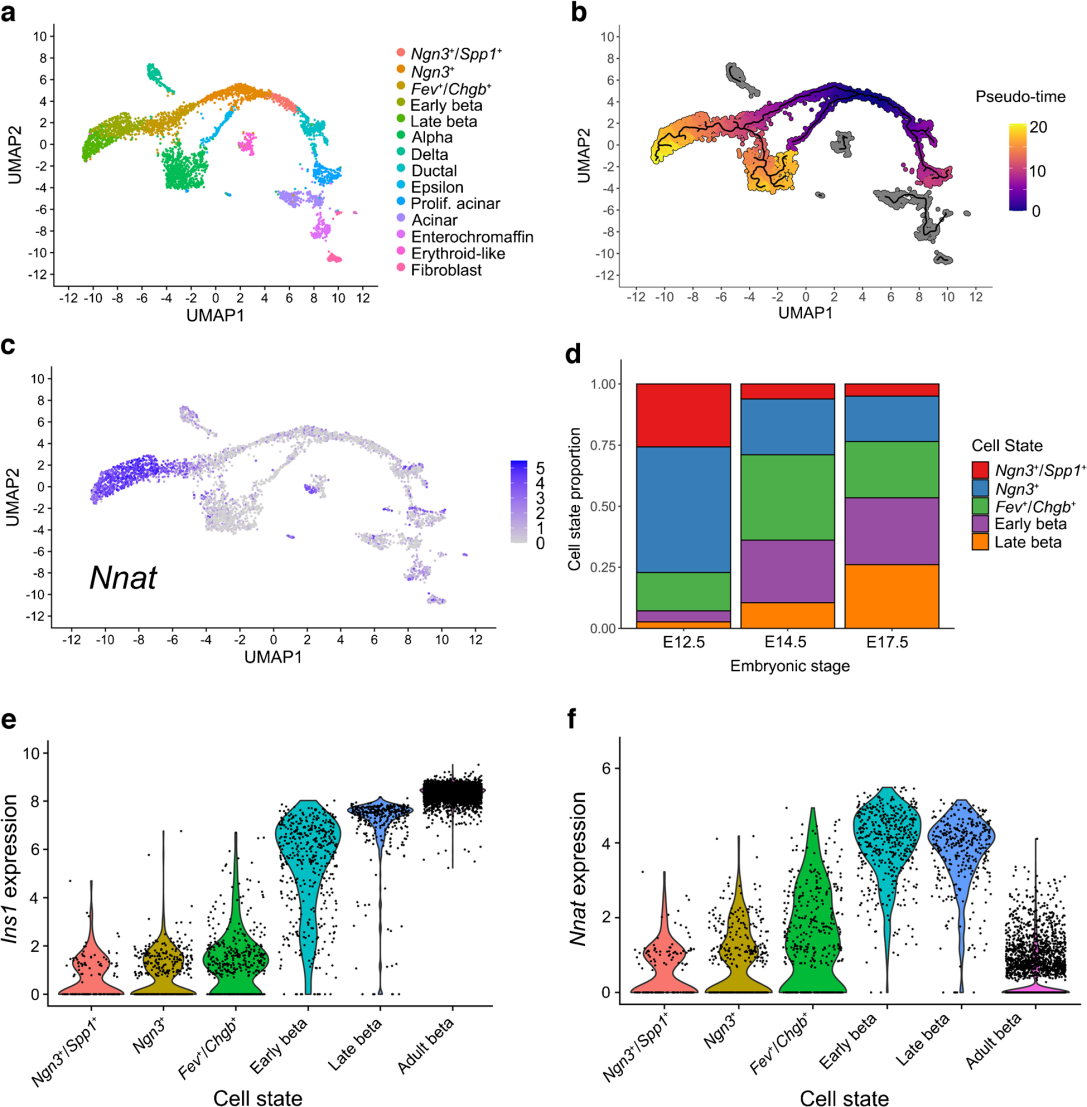
*Nnat* expression is a marker for late-stage beta cell differentiation during islet development. (**a**–**c**) UMAP projection of scRNA-seq data from embryonic mouse islets (E12.5–E17.5), with cells labelled by their cellular state (**a**), pseudo-time (scale bar represents abstract unit of time) (**b**) and corresponding *Nnat* expression (log-normalised expression level) (**c**). (**d**) Changes in the distribution of beta cell precursors from E12.5 to E17.5. (**e**, **f**) Violin plots showing the fluctuations in *Ins1* (**e**) and *Nnat* (**f**) expression (both log-normalised expression level) from E12.5 to adulthood in the beta cell development trajectory. Prolif., proliferating; UMAP, uniform manifold approximation and projection

Reanalysis of published scRNA-seq datasets from primary mouse beta cells at embryonic (embryonic day 17.5 [E17.5]) [39, 40] and adult [5, 41, 42] stages identified *Nnat* as amongst the most highly variable genes between individual cells (ESM Table 1). Pseudo-time analysis of embryonic (E12.5 to E17.5) mouse beta cells [39] clearly demarcated the differentiation of beta cells through embryonic cell states, with late embryonic (E17.5) beta cells overlapping with high levels of *Nnat* expression (Fig. 1a–c, ESM Figs 2a–f, 3a–k). Analysis of the proportions of different beta cell progenitors revealed that *Nnat*^+^ cells become more prominent at later stages of development (Fig. 1d). Integrating cells from the embryonic beta cell trajectory with adult beta cells [41, 42] allowed us to evaluate the progressive change in beta cell markers and *Nnat* expression from development to maturity (Fig. 1e,f, ESM Fig. 4a–i), and revealed large increases in *Ins1* expression alongside *Nnat* downregulation in most beta cells. Nevertheless, a considerable number of adult beta cells (with high expression of *Ins1*/*Ins2*) also expressed *Nnat* (Fig. 1f). Thus, *Nnat* appears to mark late-stage beta cell differentiation, peaking in expression around E17.5 and then gradually being downregulated across most beta cells in adulthood.

**Fig. 2:**
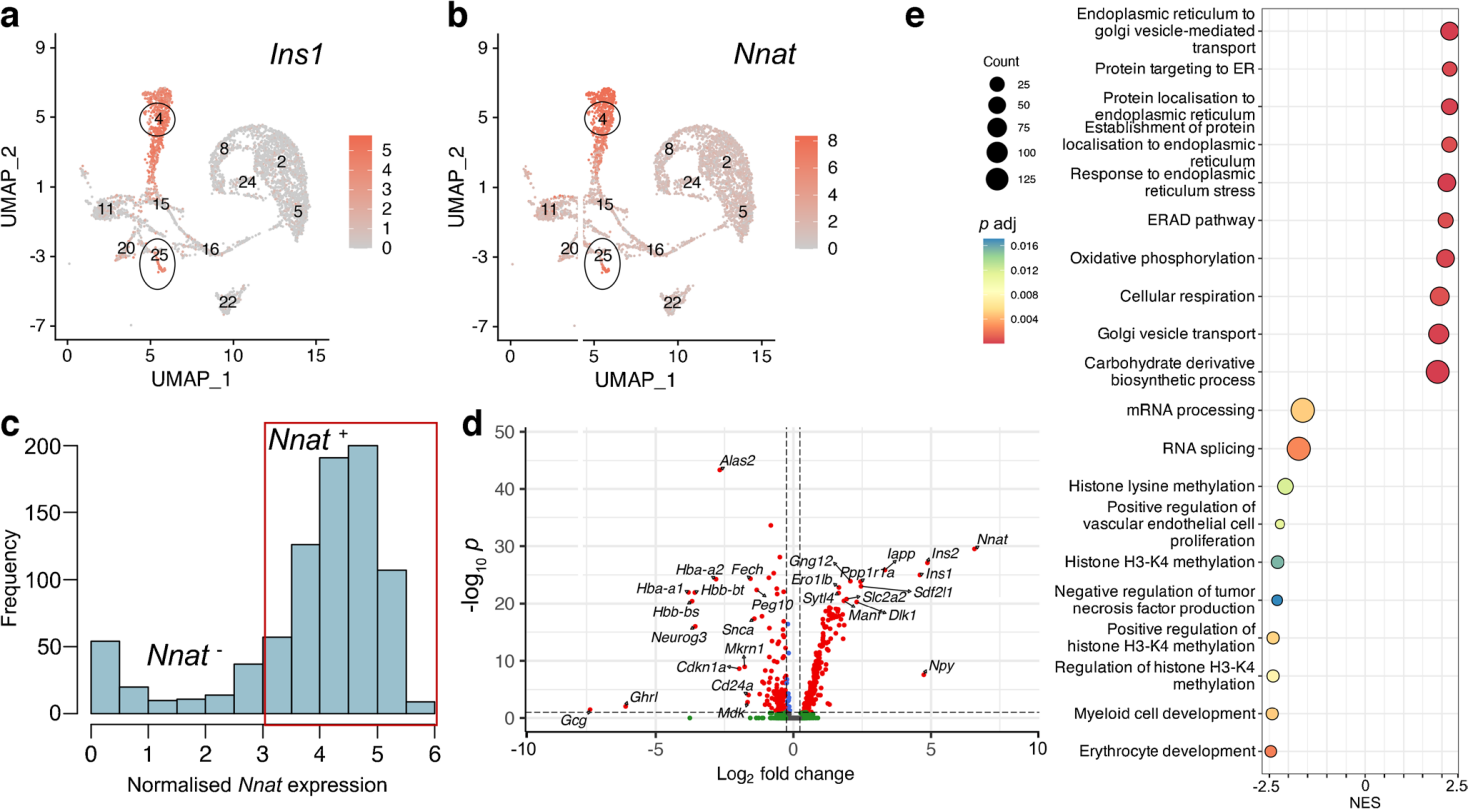
Transcriptomic analyses of islet cells at E17.5 reveal *Nnat*^+^ cells to be a more differentiated beta cell subcluster. (**a**, **b**) Islet cells plotted in the UMAP space and coloured by *Ins1* (**a**) and *Nnat* (**b**) expression, respectively. Individual cellular clusters are labelled. Beta cell clusters are highlighted with black circles. (**c**) Distribution of *Nnat* expression among beta cell clusters, along with the expression range used in defining *Nnat*^+^ beta cells (highlighted by the red rectangle). (**d**) Volcano plot showing the log_2_ fold change in gene expression between *Nnat*^+^ and *Nnat*^−^ beta cells against FDR-corrected *p* values. Genes with FDR<0.1 are coloured red. Top ten upregulated and downregulated genes are labelled by gene name. (**e**) GSEA of differentially expressed genes between *Nnat*^+^ and *Nnat*^−^ beta cells (using gene ontology-biological process [GO-BP] terms), showing the top ten upregulated and downregulated processes. The normalised enrichment score (NES) is plotted along the *x*-axis. Circle diameters are proportional to the number of genes in each process, with the colours defining statistical significance. UMAP, uniform manifold approximation and projection

In light of the abundance of *Nnat*^+^ beta cells during late embryogenesis, we further evaluated the E17.5 dataset [39] and again identified two beta cell populations with distinct levels of *Nnat* expression (Fig. 2a–c, ESM Fig. 5a–f). Embryonic *Nnat*^*+*^ beta cells were enriched for *Ins1*, *Ins2*, *Nkx6.1*, *Pdx1*, *Ucn3*, *Slc2a2*, *Iapp*, *Ero1b*, *G6pc2*, *Dlk1* and *Npy* expression compared with *Nnat*^−^ beta cells which had higher *Neurog3*, *Pax4*, *Gcg*, *Arx* and *Ghrl* expression, indicating that the *Nnat*^*+*^ beta cells were more fully differentiated (Fig. 2d, ESM Table 2). *Nnat*^*+*^ beta cells were also de-enriched for *Cd24a* (Fig. 2d, ESM Table 2). Gene set enrichment analysis (GSEA) revealed upregulation of processes related to protein synthesis, transport from endoplasmic reticulum (ER), ER stress, as well as oxidative phosphorylation and carbohydrate metabolism, with downregulated processes including mRNA processing, splicing and histone methylation (Fig. 2e). Indeed, a separate cell clustering analysis [46] identified *Nnat* as a highly differentially expressed gene between two beta cell clusters at both the late embryonic [39, 40] and adult [5] stages (ESM Figs 6a–h, 7a–g, ESM Table 3).

**Fig. 3:**
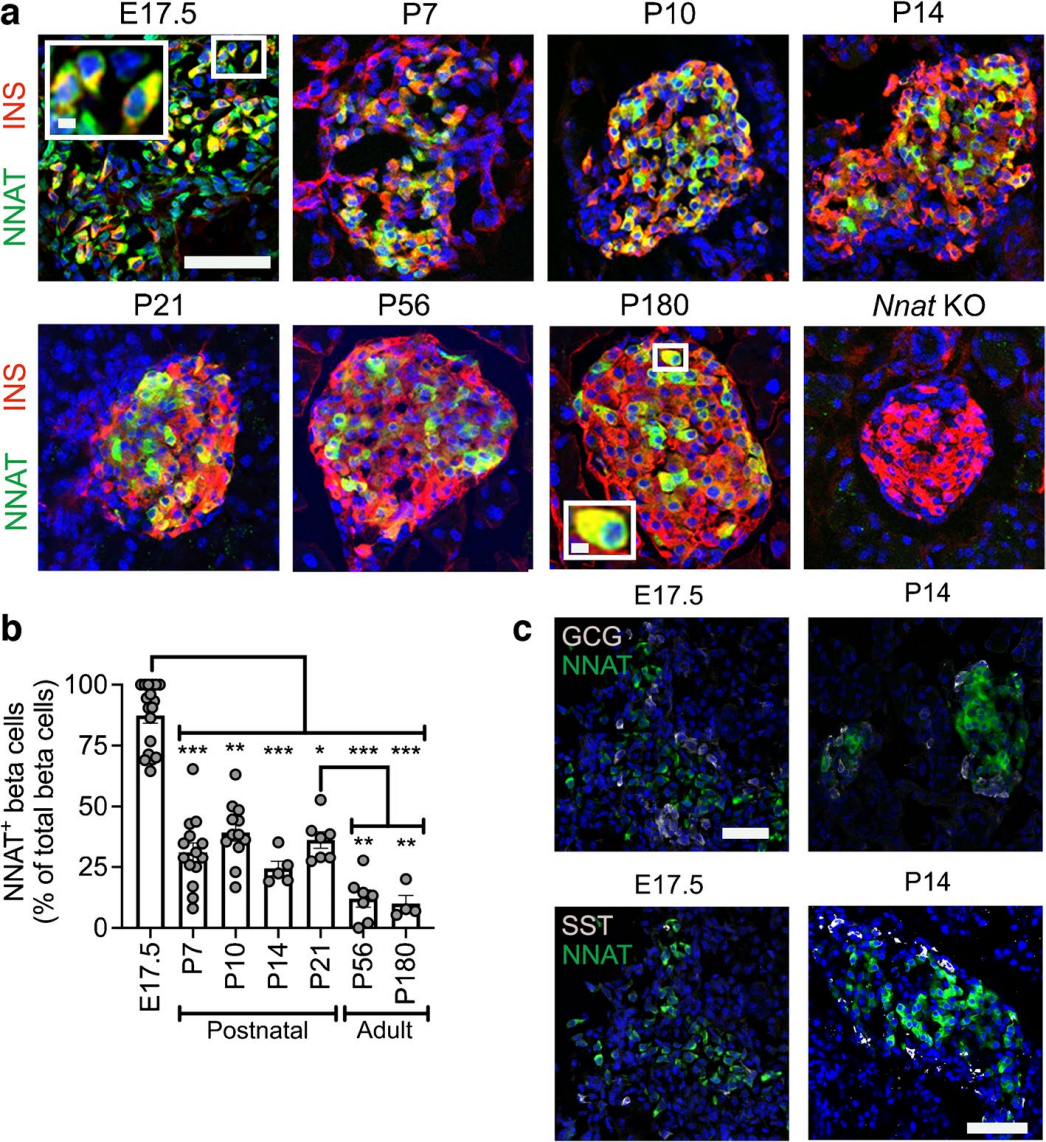
A subpopulation of NNAT^+^ beta cells develops during the early postnatal period in mice. (**a**) Representative confocal microscopy of pancreatic cryosections from wild-type mice on a C57BL/6J background of developmental stages from E17.5 through the postnatal period into adulthood. Sections were immunostained with antibodies against endogenous NNAT (green) and insulin (INS, red). Nuclei are visualised with DAPI and sections from P56 mice with constituent deletion of *Nnat* were used as an immunostaining control. Scale bar, 100 μm (inset, 10 μm). (**b**) Quantification of NNAT^+^ beta cells from images shown in (**a**), expressed as NNAT/INS co-positive cells as a percentage of total INS-positive cells (*n*=4–15 mice per timepoint, Kruskal–Wallis test with Dunn’s multiple comparisons). (**c**) Representative confocal microscopy of pancreatic cryosections as in (**a**) from E17.5 and P14 mice (*n*=18 and *n*=5 mice, respectively) immunostained with antibodies against endogenous NNAT (green) and GCG or SST (both grey). Scale bar, 100 μm. Representative images from three independent experiments and breeding pairs. *p<0.05, **p<0.01, ***p<0.001. GCG, glucagon; KO, knockout; SST, somatostatin

We confirmed some of these findings at the protein level using immunofluorescence in postnatal and adult mice (Fig. 3a,b). We noted that NNAT protein expression is highly dynamic in beta cells across the mouse islet throughout the postnatal stage, transitioning from expression throughout the majority (90.4±3.2%, *n*=15 mice) of beta cells at late embryogenesis to a subset (24.4±3.0%, *n*=5 mice) of beta cells by postnatal day 14 (P14) (Fig. 3a,b). NNAT^+^ beta cells persisted throughout the islet into adulthood (Fig. 3a,b). NNAT expression was not apparent in other mouse islet cell types, including alpha and delta cells, at P14. However, NNAT expression was detectable in a small number of alpha and delta cells at E17.5 (Fig. 3c).

### NNAT immunoreactivity is detected in a subset of beta cells in human islets and NNAT deficiency in human beta cells blunts GSIS

**Fig. 4:**
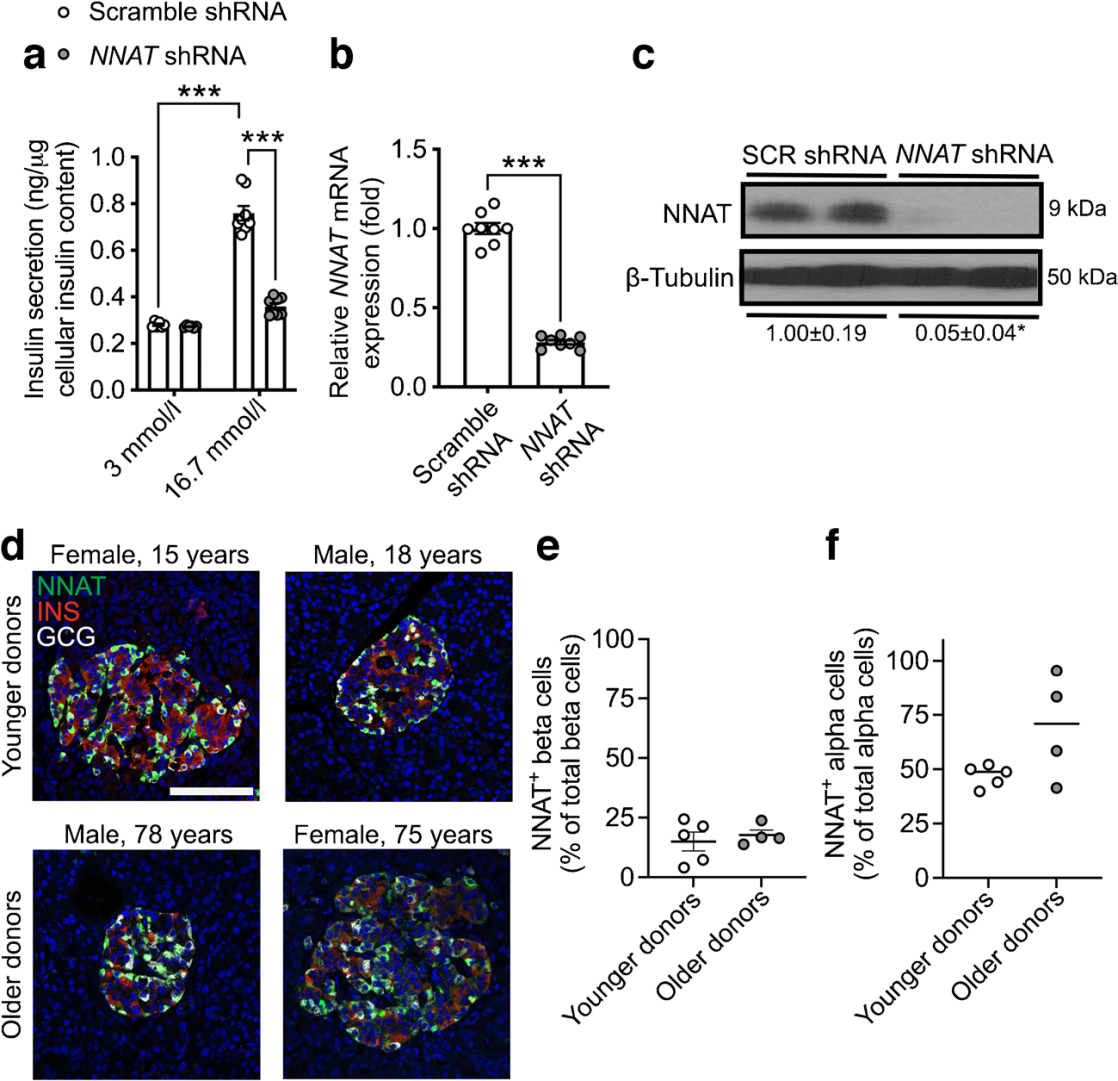
NNAT is expressed in a subset of human beta cells and NNAT deficiency in human beta cells reduces insulin secretion. (**a**) GSIS analysis at low (3 mmol/l) and high (16.7 mmol/l) glucose in human EndoC-βH1 beta cells following 72 h of lentiviral-mediated shRNA knockdown of NNAT with data expressed as insulin secreted into culture media as a percentage of total cellular insulin content (*n*=8 independent cultures per group, two-way ANOVA with Sidak’s multiple corrections test). (**b**, **c**) RT-PCR (*n*=8, unpaired Student’s *t* test) and western blot (*n*=4, Mann–Whitney *U* test) analysis of NNAT expression in EndoC-βH1 beta cells after transient *NNAT* silencing as in (**a**). (**c**) Western blotting analysis of NNAT protein levels shown via a representative blot of two independent experiments. β-Tubulin was used as a loading control. Mean values for band intensities in multiple experiments quantified by densitometry are shown below the panel, expressed relative to scramble (SCR) shRNA controls. (**d**) Representative confocal microscopy of human pancreatic cryosections from younger (15.6±0.9 years, *n*=5) and older (71.0±3.9 years, *n*=4) donors. Sections were immunostained with antibodies against endogenous NNAT (green), insulin (INS, red) and glucagon (GCG, grey). Donor sex and age are indicated in the text. Nuclei are visualised with DAPI. Scale bar, 100 μm. (**e**, **f**) Quantification of NNAT^+^ beta (**e**) and alpha (**f**) cells from images shown in (**d**), expressed as NNAT/INS or NNAT/GCG co-positive cells as a percentage of total INS-positive or GCG-positive cells. *p<0.05, ***p<0.001

While the role of NNAT has previously been examined in mouse beta cells [35], no data currently exist for human beta cells. We therefore next compared the functionality of human NNAT^+^ and NNAT^−^ beta cells. Transient silencing of *NNAT* in human EndoC-βH1 beta cells completely abrogated glucose-stimulated insulin release (Fig. 4a–c). As in mouse beta cells [35], NNAT interacted in cellulo with subunits of the SPC, SEC11A, SPCS1, SPCS2 and SPCS3 (ESM Fig. 8a), likely via SPCS1 (ESM Fig. 8b).

Heterogeneous expression of NNAT in human islet cells was also observed in whole pancreatic sections from multiple donors. Thus, NNAT was expressed in a subset of beta cells in both younger (15.6±0.9 years, 15.0±4.0%, *n*=5) and older (71.0±3.9 years, 17.7±2.1%, *n*= 4) donors (Fig. 4d,e, ESM Fig. 9a, ESM Table 4, ESM Human Islet Checklist). Interestingly, NNAT was also expressed in a large fraction of human alpha cells (but not in delta cells, Fig. 4d,f, ESM Fig. 9b) in both younger (47.0±2.2%) and older donors (69.6±12.2%).

### Heterogeneous NNAT expression in beta cells during postnatal development is associated with altered CpG methylation

**Fig. 5:**
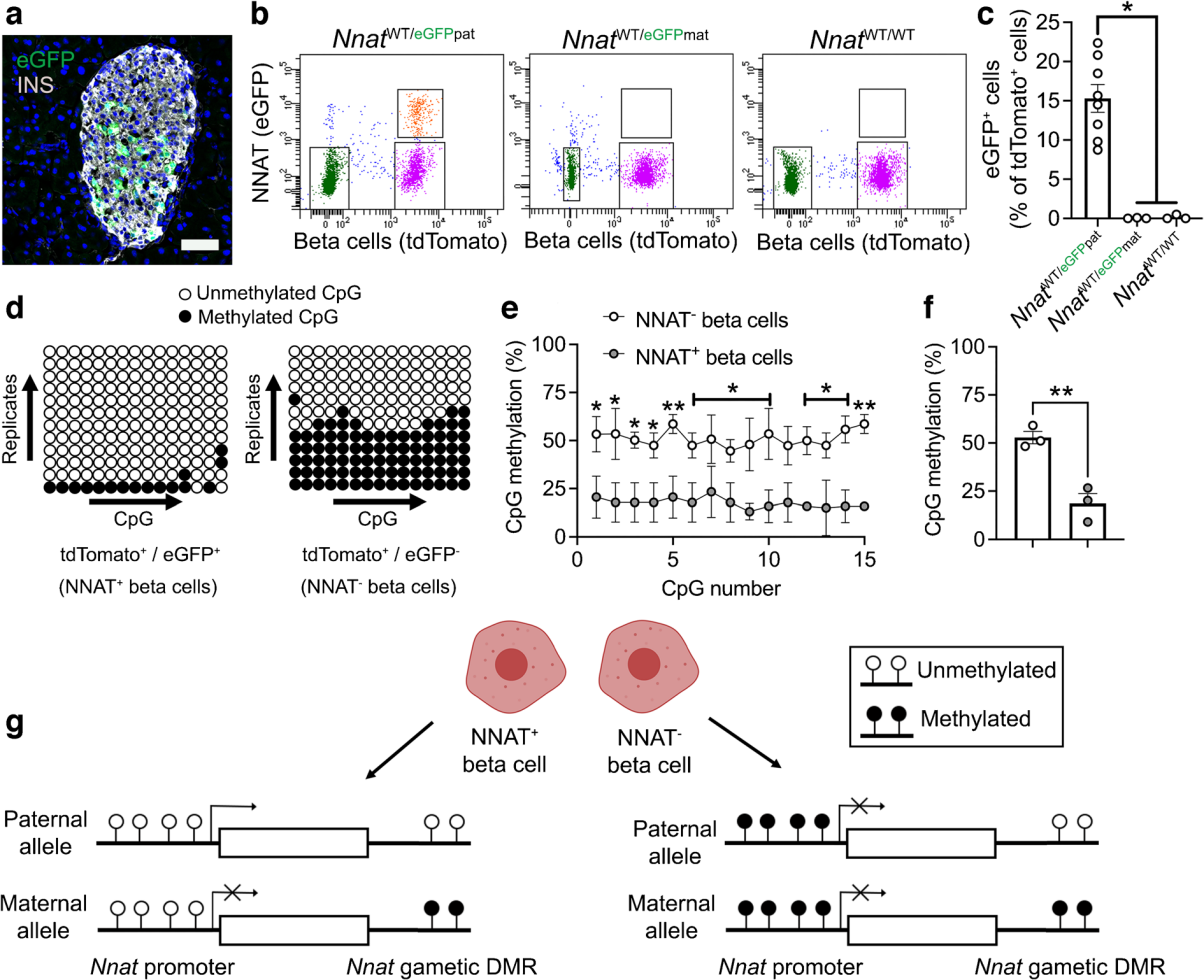
Beta cell heterogeneity of NNAT expression is associated with changes in CpG methylation at the *Nnat* promoter. (**a**) Representative confocal microscopy of pancreatic cryosections from P56 mice with *Nnat*-driven eGFP expression from the paternal allele (*Nnat*^WT/eGFPpat^) (*n*=7 mice). Sections were immunostained with antibodies against eGFP (green) and insulin (INS, grey). Nuclei are visualised with DAPI. Scale bar, 50 μm. (**b**) FACS separation of dispersed primary islet cells from reporter mice with insulin-driven expression of tdTomato (to label beta cells) and *Nnat*-driven eGFP expression from the paternal (*Nnat*^WT/eGFPpat^) or maternal *(Nnat*^WT/eGFPmat^) allele or wild-type (*Nnat*^WT/WT^) at this locus (representative FACS plot of the dispersed islet preparation from a single mouse shown) (*n*=8, 3 and 3 mice per genotype, respectively, Kruskal–Wallis test with Dunn’s multiple comparisons). (**c**) Quantification of data in (**b**) expressed as percentage of eGFP/tdTomato co-positive primary islet cells. (**d**) Representative bisulphite analysis of CpG methylation at the *Nnat* promoter in FACS-purified islet cell populations from (**b**) (*n*=3 *Nnat*^WT/eGFPpat^ mice with paternally expressed *Nnat*-driven eGFP, *n*≥12 clones each). Closed circles, methylated CpG; open circles, unmethylated CpG. (**e**, **f**) Quantification of data in (**d**) expressed as percentage CpG methylation across the *Nnat* promoter at individual CpGs (**e**) and across the entire *Nnat* promoter (**f**) (both paired Student’s *t* test). (**g**) Schematic summarising level of CpG methylation at the *Nnat* promoter and gametic DMR in NNAT^+^ vs NNAT^−^ beta cells (created with BioRender.com). Representative image in (**a**) and bisulphite analyses in (**d**) used experimental mice from three independent experiments and breeding pairs. *p<0.05, **p<0.01

To extend the findings above using an orthogonal approach, and to facilitate subsequent functional studies, we utilised a Bacterial Artificial Chromosome (BAC) reporter mouse line expressing eGFP under the control of the *Nnat* promoter and upstream enhancers (ESM Results, ESM Fig. 10a–e). Aligning with studies of the endogenous gene (Figs 1, 2, 3 and 4), adult reporter mice expressed eGFP in a subpopulation (~15% of total) of beta cells (Fig. 5a).

To isolate and purify beta cells based on NNAT levels, we crossed *Nnat*-eGFP reporter mice to animals expressing *Cre* recombinase under the RIP [43] and to transgenic mice expressing a tdTomato reporter downstream of a *lox*P-flanked stop codon [44]. Dispersion of primary adult islets into single cells, and subsequent FACS analysis, verified the presence of NNAT^+^ and NNAT^−^ beta cells, with the NNAT^+^ fraction composing 15.3±1.8% (*n*=8 mice) of the total beta cell compartment (Fig. 5b,c, ESM Figs 11a,b, 12a–f). Collection of FACS-purified NNAT^+^ and NNAT^−^ beta cells and subsequent bisulphite sequencing analysis revealed that CpG methylation at the gametic DMR (known to control monoallelic *Nnat* expression; Introduction) was unchanged (ESM Fig. 11c–e). Nevertheless, CpG methylation at the *Nnat* promoter was significantly altered across this genomic region, with minimal CpG methylation observed in NNAT^+^ beta cells (Fig. 5d–f).

Recent whole-genome methylation analysis in mouse germ cells has revealed that the *Nnat* promoter region is unmethylated in sperm, and this was confirmed by targeted bisulphite sequencing analysis (ESM Fig. 11f–h), whereas it is fully methylated in oocytes [47]. Thus, the ‘classical’ imprinting of *Nnat* is unaltered between beta cell subtypes with near binary (‘on/off’) expression of NNAT. However, and overlaying this control, a second DMR at the *Nnat* promoter within the non-imprinted allele dictates beta cell subtype specificity of expression (Fig. 5g).

### The de novo DNA methyltransferase DNMT3A establishes NNAT beta cell subtype specificity

**Fig. 6:**
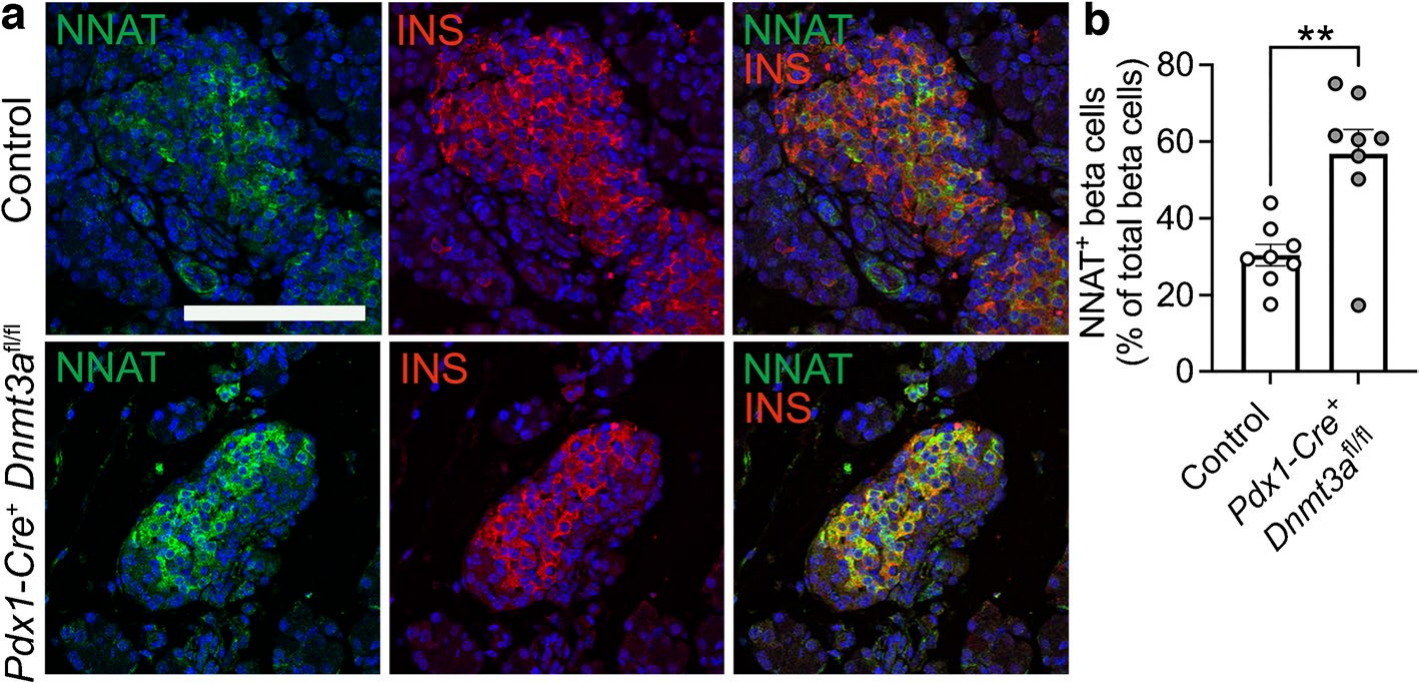
Postnatal restriction of NNAT in a subset of beta cells is at least partially driven by the de novo methyltransferase DNMT3A. (**a**) Representative confocal microscopy of pancreatic cryosections from mice with conditional deletion of DNMT3A under the control of the *Pdx1* promoter (*Pdx1*-*Cre*^*+*^* Dnmt3a*^fl/fl^) vs control (*Pdx1*-*Cre*^*−*^* Dnmt3a*^fl/fl^) mice at P6. Sections were immunostained with antibodies against endogenous NNAT (green) and insulin (INS, red). (**b**) Quantification of NNAT^+^ beta cells from images shown in (**a**), expressed as NNAT/INS co-positive cells as a percentage of total INS-positive cells. Scale bar, 50 μm (*n*=8 mice per genotype, unpaired Student’s *t* test, **p<0.01). Nuclei are visualised with DAPI

To ask whether this apparent transition in *Nnat* expression in the early postnatal period is driven by de novo CpG methylation, we assessed endogenous NNAT beta cell immunoreactivity in postnatal mice conditionally deleted for the methyltransferase DNA methyltransferase 3 alpha (DNMT3A) at the pancreatic progenitor stage (using *Pdx1*-*Cre*) [6]. NNAT staining in control mice at P6 demonstrated expression in a subpopulation of beta cells, whereas deletion of DNMT3A resulted in a loss of this heterogenous expression across the islet (Fig. 6a,b, ESM Fig. 13a). These findings demonstrate that de novo methylation is likely to drive NNAT restriction across the beta cell complement in the first few days of postnatal life.

### NNAT^+^ and NNAT^−^ beta cells have distinct transcriptional signatures

**Fig. 7:**
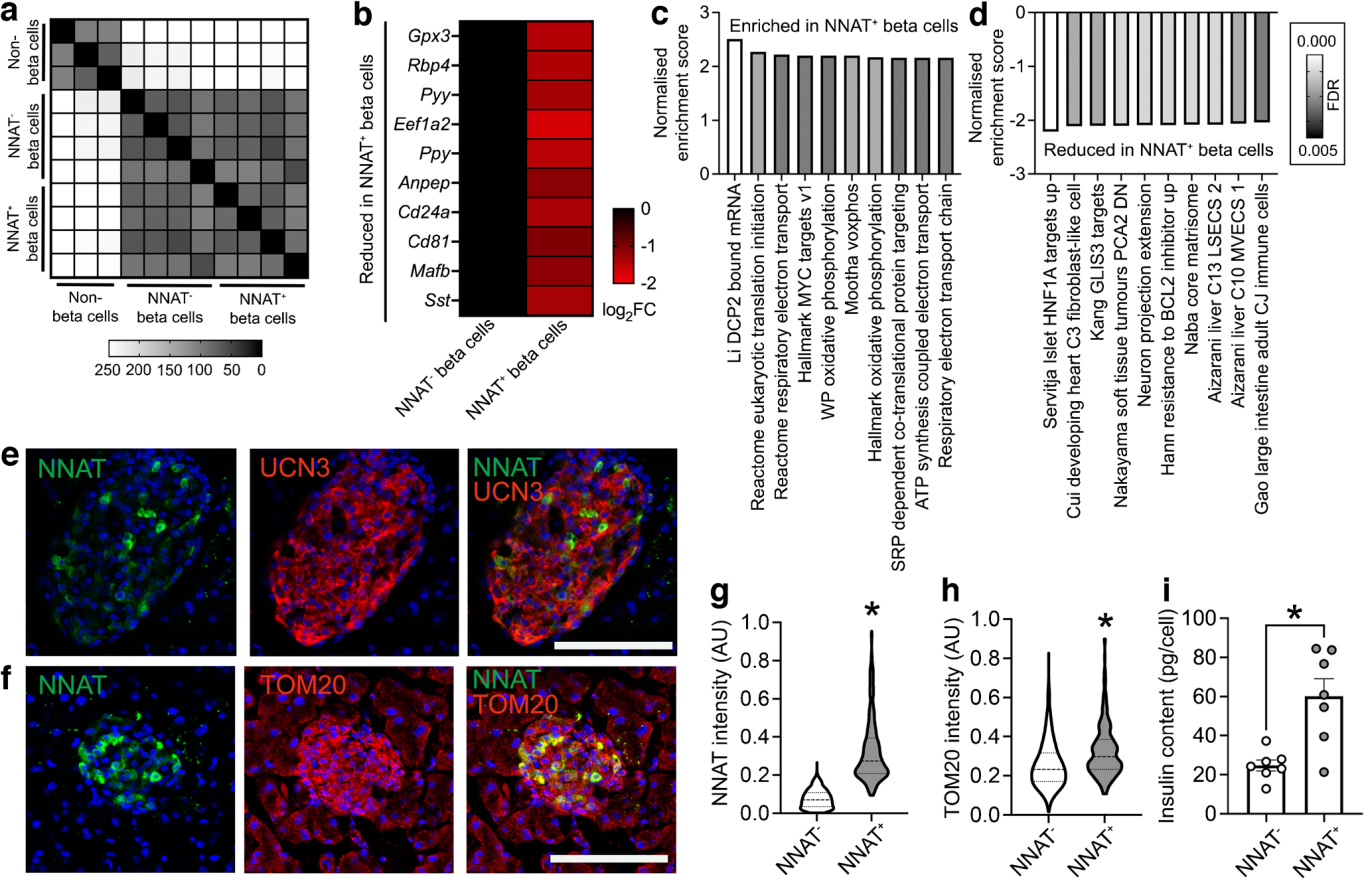
NNAT^+^ adult beta cells are transcriptionally distinct and have significantly higher insulin content. (**a**) Correlation matrix of differentially expressed genes between NNAT^+^ and NNAT^−^ beta cells as assessed by RNA-seq analysis (*n*=4 FACS-purified populations from individual mouse islet preparations). Scale bar (0–255) gives an integer provided to the R function where the darker colour corresponds to higher sample correlation. (**b**) Heatmap of the top ten most differentially expressed genes reduced in NNAT^+^ beta cells compared with NNAT^−^ beta cells. (**c**, **d**) GSEA showing categories significantly enriched (**c**) and reduced (**d**) in NNAT^+^ (vs NNAT^−^) beta cells. (**e**, **f**) Representative confocal microscopy of pancreatic cryosections from P56 (8-week-old) wild-type mice on a C57BL/6J background immunostained with antibodies against endogenous NNAT (green) and UCN3 (red, **e**) or TOM20 (red, **f**). Scale bar, 100 μm. Nuclei are visualised with DAPI. Representative images from three independent experiments and breeding pairs (**g**, **h**). Quantification of NNAT (**g**) and TOM20 (**h**) staining in NNAT^−^ and NNAT^+^ beta cells (INS^+^) from images shown in (**f**), expressed as mean intensity of the NNAT or TOM20 channel (*p<0.05, paired Student’s *t* test, 1329 NNAT^−^ and 295 NNAT^+^ beta cells from 15 islets, *n*=3 mice). (**i**) Insulin content assessed in NNAT^+^ and NNAT^−^ beta cells (*p<0.05, *n*=7 FACS-purified populations from individual mouse islet preparations, Wilcoxon matched-pairs signed rank test). AU, arbitrary units; FC, fold change

In contrast to the embryonic populations described above, RNA-seq revealed that FACS-sorted adult (8 weeks) NNAT^+^ and NNAT^−^ beta cells displayed high transcriptional overlap and were similar to each other when compared with non-beta endocrine cells (Fig. 7a). Major beta cell identity markers such as *Ins1*, *Ins2*, *Mafa*, *Slc2a2* (*Glut2*) and *Nkx6.1* were not differentially expressed (ESM Table 5).

Nevertheless, we identified 241 (1.8%) and 79 (0.6%) genes that were significantly down- and upregulated in NNAT^+^ vs NNAT^−^ beta cells, respectively (>2-fold, false discovery rate [FDR]<0.01). Differentially expressed genes included several markers of non-beta cell islet lineages (*Pyy*, *Ppy*, *Mafb*, *Sst*) that were lower in the NNAT^+^ beta cell fraction, genes linked with beta cell heterogeneity and plasticity (*Gpx3*, *Rbp4*), the beta cell immaturity marker *Cd81* [20] and the *Cd24a* antigen [21] (Fig. 7b). Interestingly, *Npy* was enriched in NNAT^+^ beta cells (ESM Table 5), consistent with our observations at late embryogenesis (E17.5, Fig. 2d). We did not observe any clear differences between NNAT^+^ and NNAT^−^ beta cells in the expression of other imprinted genes known to be functional in the beta cell (*Plagl1*/*ZAC*, *Dlk1*, *Rasgrf1*, *Cdkn1c*, *Grb10* and *Gtl2*/*MEG3*), nor changes in ‘disallowed’ genes whose expression is known to be restricted in mature functional beta cells (*Hk1*, *Mct1* [*Slc16a1*] and *Ldha*) [48] (ESM Table 5). Likewise, no differences were apparent in the levels of transcripts encoding proteins previously demonstrated by others to mark specific beta cell subpopulations, such as *Flattop*/*Cfap126* [16], *CD9* and *ST8SIA1* [10], *Cd63* [23], ‘virgin’ beta cells (via *Ucn3*) [24] or those differentially expressed in beta cells implicated in the control of Ca^2+^ dynamics (‘hubs’ [14], and ‘leaders’ [15, 26]).

GSEA did, however, reveal enrichment of pathways in NNAT^+^ beta cells including translation initiation, the electron transport chain (ETC), oxidative phosphorylation and signal recognition particle (SRP)-dependent co-translational protein targeting (Fig. 7c). Pathways involving genes associated with hepatocyte nuclear factor 1 alpha (HNF1A) and GLI-similar family zinc finger 3 (GLIS3) targets were reduced in the NNAT^+^ beta cell fraction (Fig. 7d). NNAT^+^ beta cells displayed higher levels of the late maturation marker urocortin 3 (UCN3) (Fig. 7e, 0.357±0.009 in NNAT^+^ vs 0.264±0.004 arbitrary units in NNAT^−^, **p<0.01, 290 NNAT^+^/1876 NNAT^−^ beta cells from 13 islets/*n*=3 mice) and a small but significant increase in translocase of outer mitochondrial membrane 20 (TOM20) immunostaining, consistent with a higher mitochondrial volume or number (Fig. 7f–h). Furthermore, FACS-sorted primary NNAT^+^ beta cells had a significantly higher insulin content than NNAT^−^ cells (Fig. 7i).

### The NNAT^+^ beta cell population shows impaired glucose-stimulated Ca^2+^ dynamics and is de-enriched for highly connected ‘hub’ cells

To determine whether NNAT may influence beta cell connectivity and membership of the ‘hub’ cell subgroup [14], we studied glucose-induced Ca^2+^ dynamics in *Nnat*-deficient (*Nnat*^+/−p^) islets [35]. Ca^2+^ increases in response to high (11 mmol/l) glucose were significantly higher in *Nnat*^+/−p^ than control islets, and while the mean Pearson’s coefficient of correlation and proportion of highly connected ‘hub’ cells were not significantly different between *Nnat*-deficient and control islets (ESM Results, ESM Fig. 14a–d), wild-type ‘hub’ cells were significantly more connected than *Nnat*-deficient ‘hub’ cells (ESM Fig. 14e,f). Thus, NNAT is a marker of less well-connected cells.

**Fig. 8:**
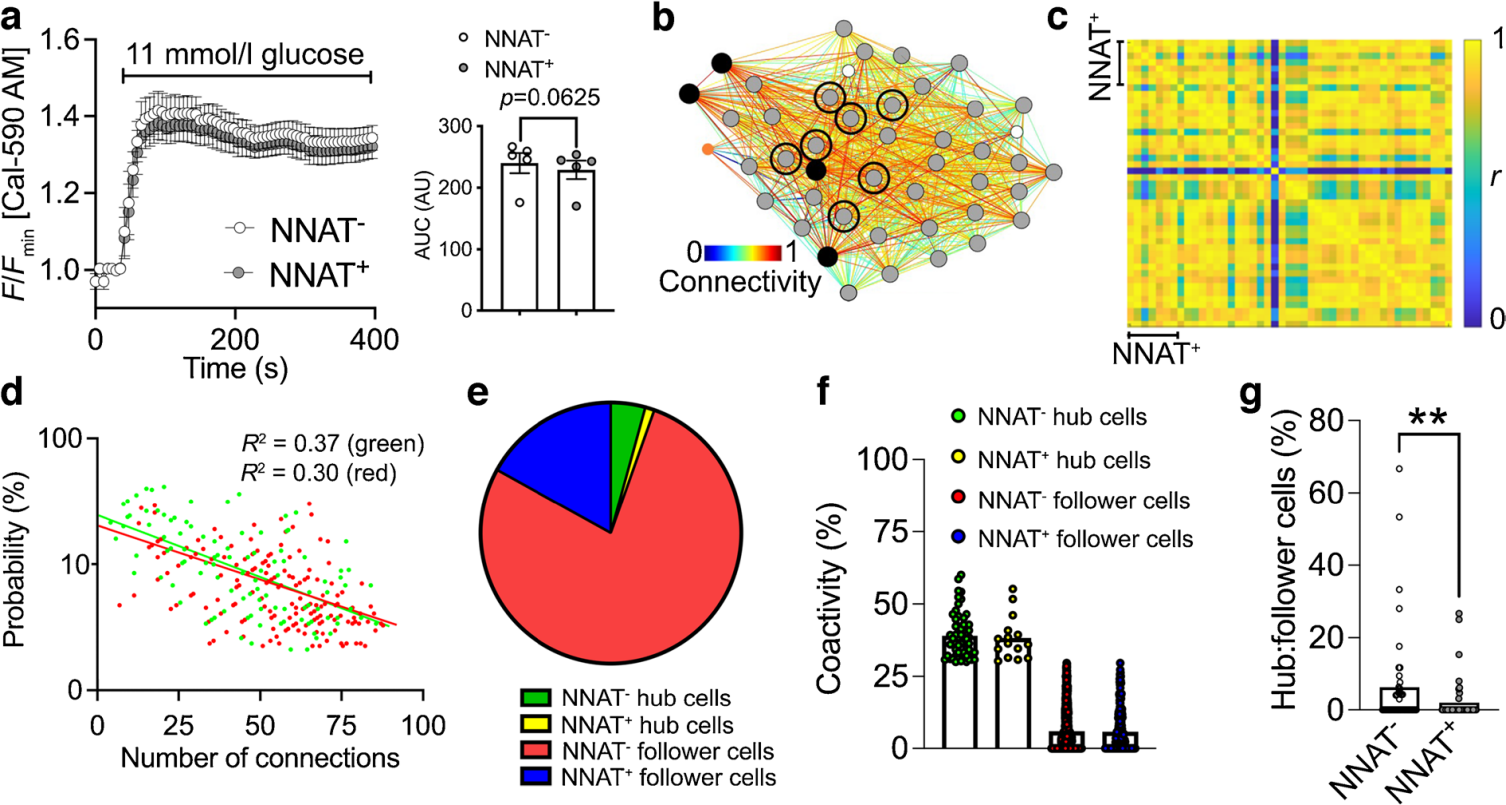
NNAT^+^ beta cells show altered glucose-induced Ca^2+^ dynamics and are de-enriched for highly connected ‘hub’ cells within individual islets. (**a**) Ca^2+^-bound Cal-590 AM fluorescence in response to high glucose (11 mmol/l) in NNAT^−^ and NNAT^+^ cells from primary islets from *Nnat*^WT/eGFPpat^ reporter mice expressed as normalised intensity over time (*F*/*F*_min_) (*n*=61 islets total from five mice per genotype; quantification of AUC on the right, *p*=0.063, Wilcoxon matched-pairs signed rank test). (**b**) Representative Cartesian map of beta cells with colour-coded lines connecting cells according to the strength of coactivation (colour-coded *R* values from 0 to 1, blue to red). Beta cells are represented by differently coloured nodes depending on their coactivity with the other beta cells, where black nodes indicate coactivity with ≥80% of the remaining beta cells, while grey, white and orange nodes represent coactivity with ≥60%, ≥40% and <40%, respectively. Nodes circled with a solid black line indicate NNAT^+^ cells. (**c**) Representative heatmaps depicting connectivity strength (*r*) of all cell pairs (colour-coded *r* values from 0 to 1, blue to yellow). (**d**) Log–log graphs of beta cell–beta cell connectivity distribution. NNAT^+^ cells are represented by green circles while NNAT^−^ cells are represented by red circles (45 islets total using primary islet preparations, each from an individual *Nnat*^WT/eGFPpat^ reporter mouse). (**e**) Categorisation of beta cells based on data from (**d**). (**f**) Percentage coactivity of beta cells between all cells in identified ‘hub’ and ‘follower’ cells. (**g**) The proportion of cells designated as ‘hub’ vs ‘follower’ cells in both the NNAT^−^ and NNAT^+^ populations assessed in each of 45 islets (**p<0.01, Wilcoxon matched-pairs signed rank test). Analyses in panels (**a**–**g**) obtained from three independent experiments and three different breeding pairs of experimental mice. AU, arbitrary units

We further explored this question using islets from *Nnat*-eGFP reporter mice, using the red-shifted calcium probe Cal-590 AM [49]. *Nnat*-GFP^+^ and *Nnat*-GFP^−^ cells responded similarly to challenge with high (11 mmol/l) glucose (Fig. 8a). Beta cell–beta cell connectivity (Fig. 8b) was not different between NNAT^+^ and NNAT^−^ populations and, considered across the whole population, the mean Pearson’s coefficient of correlation was 0.87±0.01 (Fig. 8c).

Of all cells examined, 4.27% were NNAT^−^ ‘hub’ cells (Ca^2+^ responses with at least 30% of all cells, Fig. 8d) connected to an mean of 39.0% of all beta cells (Fig. 8e,f). The fraction of these (1.15%, Fig. 8e) identified as NNAT^+^ ‘hub’ cells had an mean of 38.3% coactivity with other cells (Fig. 8f). However, when considered within each individual islet, a significantly lower ratio of ‘hubs’ to ‘followers’ was observed in NNAT^+^ vs NNAT^−^ cells (Fig. 8g). Overlap with ‘leader’ cells [15, 25] was not explored in the absence of readily identifiable Ca^2+^ waves.

## Discussion

We describe here a novel subgroup of beta cells characterised by transient methylation of a second DMR (but not the gametic DMR) in the *Nnat* locus. This is consistent with previous findings that methylation at gametic DMRs is thought to be relatively stable [50], whereas ‘secondary’ imprinting regions have been shown to be more sensitive to nutrient- or physiology-based changes [6, 51]. We have previously described the importance of *Nnat* for beta cell insulin content and secretion [35] and therefore hypothesise that NNAT^+^ and NNAT^−^ cells display differences in secretory function. Correspondingly, transcriptomic analysis of purified adult NNAT^+^ and NNAT^−^ beta cells demonstrated that the NNAT^+^ fraction is enriched for functional pathways including translation initiation, ETC/oxidative phosphorylation pathways and co-translational protein ER membrane targeting.

Extending these studies to humans, NNAT deficiency in human EndoC-βH1 beta cells severely blunted GSIS, likely via inhibition of signal peptidase-mediated processing [35]. We show here that E17.5 *Nnat*^+^ beta cells are enriched for expression of the SPC and translocon apparatus (*Spcs1*, *Spcs2*, *Sec11a*, *Sec11c*, *Sec61*) and that the SPC/NNAT interaction is via SPCS1, and likely the means through which NNAT influences cellular insulin content. Importantly, we demonstrate that heterogeneous expression of NNAT is also a feature of the adult human pancreatic islet. Finally, NNAT^+^ cells were de-enriched for highly connected ‘hubs’ and, thus, are likelier to belong to the ‘follower’ population [14]. Interestingly, Ca^2+^ responses to 11 mmol/l glucose were significantly higher in *Nnat*-deficient vs wild-type mouse islets, in contrast to previous findings where differences were not observed in response to 16.7 mmol/l glucose [35]. A similar strong tendency was also seen when comparing NNAT^+^ vs NNAT^−^ cells in the same islet in *Nnat*-eGFP reporter mice, and suggests that NNAT^+^ cells are less responsive to metabolic stimulation by glucose, in line with their enrichment in a ‘follower’ subset of cells, and consistent with a role in insulin production rather than glucose detection [14, 15]. The molecular underpinnings of the weaker Ca^2+^ responses are unclear, but do not appear to involve differences in the levels of transcripts encoding *Slc2a2* (*Glut2*) or *Gck*. Moreover, the transcriptome of NNAT^+^ cells does not show enrichment for genes enriched in ‘hub’ [15] or ‘leader’ cells [25].

Interestingly, bimodal expression of *Nnat* was already clearly evident at embryonic stages, with *Nnat*^+^ beta cells enriched for markers of late-stage beta cell differentiation, as well as *Ins1* and *Ins2* mRNAs. These differences were, however, less marked in the adult islet, though CD24a [7] was more weakly expressed in the NNAT^+^ population. De-enrichment of markers of other islet cell types (and the immaturity marker *Cd81*) was also a common feature of both embryonic and adult NNAT^+^ beta cells. Interestingly, both embryonic and adult NNAT^+^ beta cells were enriched for *Npy*, suggesting that these cells were not fully matured. NNAT^+^ beta cells, however, had significantly higher insulin content, suggesting a possible, at least partial, overlap with the recently described ‘CD63^hi^’ population [23], and the ‘β_HI_’ population described in reference [7].

Our work also provides evidence that the epigenome controls the fate of specific beta cell subpopulations. CpG methylation plays a crucial role in early beta cell developmental maturation, including the silencing of ‘disallowed’ genes such as *Hk1*, *Mct1* (*Slc16a1*) and *Ldha* via DNMT3A [4, 48]. Here we show that islets transition from a state wherein the majority of beta cells are NNAT^+^ in late embryogenesis to comprising a restricted subpopulation of NNAT^+^ beta cells by P7. Whether this represents changes at the level of individual beta cells, or the turnover of the NNAT^+^ population and replacement with a largely NNAT^−^ population, was not determined, and would require investigation using other approaches, including fate mapping (lineage tracing).

Adult NNAT^+^ beta cells are virtually unmethylated at the *Nnat* promoter whereas robust promoter methylation was apparent in NNAT^−^ cells. These findings, and the fact that the *Nnat* promoter is differentially methylated between sperm and oocytes (see the Results), indicate that promoter methylation during this transition is likely to be acquired on the paternal allele (from which *Nnat* is selectively expressed via genomic imprinting). Our studies also show that specific deletion of the de novo methyltransferase DNMT3A at the pancreatic progenitor stage resulted in partial loss of NNAT-based beta cell heterogeneity. Thus, DNA methylation modulates restricted NNAT expression in specific beta cells during early maturation.

Might these findings be relevant for the pathogenesis of type 2 diabetes? Whether demethylation of the second DMR at the *Nnat* locus may occur in this disease is an interesting possibility which remains to be explored. In rodents, even mild hyperglycaemia deregulates *Nnat* expression alongside that of several other critical beta cell identity genes [52], and we describe here a significant reduction of NNAT^+^ beta cells in *db/db* mice. Moreover, altered CpG methylation is a common observation in islets from individuals with diabetes at imprinted and non-imprinted loci (reviewed in [18, 32]).

In conclusion, the present work demonstrates how an effector gene in pancreatic beta cells, *NNAT/Nnat*, is controlled at the level of the epigenome (DNA methylome), contributing to a functional hierarchy between cells. Chemical modification of the epigenome may in future provide an attractive therapeutic angle not only for beta cell replacement or regeneration [53] but also to modulate beta cell function and cell–cell connectivity in type 2 diabetes.

### Supplementary Information

Below is the link to the electronic supplementary material.

Supplementary file1 (PDF 3625 KB)

Supplementary file2 (XLSX 6698 KB)

Supplementary file3 (XLSX 449 KB)

Supplementary file4 (XLSX 356 KB)

Supplementary file5 (XLSX 7092 KB)

## Data Availability

RNA-seq data are available from the GEO under accession number GSE249659. The mass spectrometry proteomics data have been deposited to the ProteomeXchange Consortium via the PRIDE partner repository with the dataset identifier PXD048465. ImageJ scripts and CellProfiler pipelines can be made available upon reasonable request. Pseudo-time analysis scripts are available via GitHub using the link https://github.com/aosakwe/Pseudotime_Analysis_MouseIslets. Transcriptomic data for *ob/ob* mice can be obtained from a publicly available source from the Alan Attie laboratory (http://diabetes.wisc.edu/search).

## Abbreviations

DMR: Differentially methylated region
DNMT3A: DNA methyltransferase 3 alpha
E: Embryonic day
eGFP: Enhanced GFP
ER: Endoplasmic reticulum
ETC: Electron transport chain
FDR: False discovery rate
GSEA: Gene set enrichment analysis
GSIS: Glucose-stimulated insulin secretion
HTRF: Homogeneous time-resolved fluorescence
NA: Numerical aperture
NNAT: Neuronatin
P: Postnatal day
RIP: Rat insulin promoter
scRNA-seq: Single-cell RNA-seq
shRNA: Short hairpin RNA
SPC: Signal peptidase complex
TOM20: Translocase of outer mitochondrial membrane 20
UCN3: Urocortin 3
